# *Arabidopsis* HIGH PLOIDY2 Sumoylates and Stabilizes Flowering Locus C through Its E3 Ligase Activity

**DOI:** 10.3389/fpls.2016.00530

**Published:** 2016-04-20

**Authors:** Jun S. Kwak, Ga H. Son, Sung-Il Kim, Jong T. Song, Hak S. Seo

**Affiliations:** ^1^Department of Plant Science and Research Institute of Agriculture and Life Sciences, Seoul National UniversitySeoul, South Korea; ^2^School of Applied Biosciences, Kyungpook National UniversityDaegu, South Korea; ^3^Plant Genomics and Breeding Institute, Seoul National UniversitySeoul, South Korea; ^4^Bio-MAX Institute Seoul National UniversitySeoul, South Korea

**Keywords:** E3 SUMO ligase, FLC, HPY2, post-translational modification, SUMO, sumoylation

## Abstract

Flowering Locus C (FLC), a floral repressor, plays an important role in flowering. The mechanisms regulating FLC gene expression and protein function have been studied extensively; however, post-translational regulation of FLC remains unclear. Here, we identified *Arabidopsis* HIGH PLOIDY2 (HPY2) as an E3 SUMO ligase for FLC. *In vitro* and *vivo* pull-down assays showed that FLC physically interacts with HPY2. *In vitro* assays showed that the stimulation of FLC sumoylation by HPY2 was dependent on SUMO-activating enzyme E1 and -conjugating enzyme E2, indicating that HPY2 was an E3 SUMO ligase for FLC. In transgenic plants, inducible *HPY2* overexpression increased the concentration of FLC, indicating that HPY2 stabilized FLC through direct sumoylation. Flowering time in *hpy2-2* mutants was shorter than in wild-type plants under long- and short-day conditions, with a greater effect under short-day conditions, and FLC was downregulated in *hpy2-2* mutants. These data indicate that HPY2 regulates FLC function and stability at both the transcriptional and post-translational levels through its E3 SUMO ligase activity.

## Introduction

Post-translational modification is an important mechanism for the regulation of protein function and stability. To date, more than 200 types of protein modification have been reported (Castro et al., 2012). One of these protein modifications, sumoylation, targets lysine for addition of a small ubiquitin-related modifier (SUMO; Wilkinson and Henley, 2010). SUMO is a small peptide with a molecular mass of approximately 11 kDa. In yeast and animal systems, SUMO regulates various cellular processes such as stress and defense responses, nitrogen metabolism, hormone signaling, epigenetic gene expression, growth, and flowering (Hotson et al., 2003; Kurepa et al., 2003; Lois et al., 2003; Murtas et al., 2003; Miura et al., 2005, 2007a; Yoo et al., 2006; Catala et al., 2007; Lee et al., 2007; Conti et al., 2008; Park et al., 2011; Son et al., 2014; Kim D.Y. et al., 2015; Kim S.I. et al., 2015).

Although, conjugation of SUMO to target proteins can occur independently of ligases, modification of target proteins with SUMO is usually catalyzed by E3 SUMO ligases (Wilkinson and Henley, 2010). The four types of E3 SUMO ligases identified to date are RanGAP1-binding protein 2 (RanBP2), polycomb group 2 (Pc2), non-SMC element/methyl methanesulfonate sensitive 1 (NES2/MMS21), and SAP and MIZ/protein inhibitor of activated STAT (SIZ/PIAS; Johnson and Gupta, 2001; Kahyo et al., 2001; Rose and Meier, 2001; Pichler et al., 2002; Kagey et al., 2003; Verger et al., 2003). PIAS/SIZ-type E3 SUMO ligase has five domains: SAP, PINIT, SP-RING finger, SXS, and PHD (Sharrocks, 2006; Miura et al., 2007a; Garcia-Dominguez et al., 2008; Cheong et al., 2009). To date, two PIAS-type SUMO E3 ligases, SIZ1 (Miura et al., 2005, 2007a) and High Ploidy 2 (HPY2; Huang et al., 2009; Ishida et al., 2009, 2012), have been reported in *Arabidopsis*.

AtSIZ1 is involved in nutrient assimilation, hormone signaling, growth, flowering, and stress responses (Miura et al., 2005, 2007b, 2010; Yoo et al., 2006; Catala et al., 2007; Lee et al., 2007; Garcia-Dominguez et al., 2008; Jin et al., 2008; Miura and Ohta, 2010; Park et al., 2011; Son et al., 2014; Kim D.Y. et al., 2015; Kim S.I. et al., 2015). AtSIZ1 has several target proteins, including the nitrate reductases NIA1 and NIA2, INDUCER OF CBF EXPRESSION 1 (ICE1), the R2R3-type transcription factor MYB30, FLOWERING LOCUS C (FLC), SLEEPY1 (SLY1), and chromomethylase 3 (CMT3; Elrouby and Coupland, 2010; Miller et al., 2010; Miura and Hasegawa, 2010; Park et al., 2011; Zheng et al., 2012; Conti et al., 2014; Son et al., 2014; Kim D.Y. et al., 2015; Kim S.I. et al., 2015).

AtHPY2 is much smaller than AtSIZ1, comprising 249 amino acids and only a single domain (SP-RING zinc finger domain). Little is known about the role of HPY2, but studies suggest it is involved in regulating cell cycle progression, meristem development, and auxin signaling (Ishida et al., 2009; Okushima et al., 2014). AtSIZ1 and HPY2 exhibit different expression patterns, and their mutants display distinct dwarf phenotypes (Ishida et al., 2012). In addition, their reciprocal expression does not complement the single-mutant phenotypes (Ishida et al., 2012). To date, there have been no reports of sumoylation of specific target proteins by HPY2.

Flowering Locus C, a MADS-box transcription factor, participates in the control of flowering time (Samach et al., 2000; Simpson and Dean, 2002). Expression of FLC is negatively regulated by components of the autonomous pathway and by vernalization (Michaels and Amasino, 1999; Sheldon et al., 1999; He and Amasino, 2005; Krichevsky et al., 2006; Greb et al., 2007), and *FLC* transcription is modulated by epigenetic methylation (Swiezewski et al., 2009; Heo and Sung, 2011). Ubiquitination via E3 ubiquitin ligase activity of SINAT5 is thought to regulate FLC stability (Park et al., 2007). FLC function and stability are also controlled by sumoylation, although the associated E3 SUMO ligase remains unidentified (Son et al., 2014). These findings suggest that post-translational mechanisms are involved in the regulation of floral transition by FLC. However, although transcriptional control of *FLC* is relatively well-characterized, the mechanisms underlying post-translational modification of FLC remain unclear.

Here, we provide the first evidence that HPY2 functions as an E3 SUMO ligase for FLC, directly interacting with FLC to catalyze its sumoylation. Sumoylation stabilizes FLC, and loss of HPY2 causes early flowering. These findings indicate that HPY2 positively controls FLC-mediated flowering repression through FLC sumoylation.

## Materials and Methods

### Plant Materials and Growth Conditions

Columbia-background ecotype *Arabidopsis thaliana* [wild-type (WT)] and the T-DNA insertion *hpy2-2* mutant were used in this study. To sterilize the seeds, WT and *hpy2-2* mutant seeds were treated with 5% sodium hypochlorite and 0.1% Triton X-100 for 10 min. For germination and growth in medium, the seeds were thoroughly washed with distilled water and then stored at 4°C in the dark. After 4 days, the seeds were plated on Murashige and Skoog (MS) agar medium including 2% sucrose. To prepare soil-grown plants, WT and *hpy2-2* mutant seeds were directly sown into vermiculite soil. Plants, including seedlings, were grown at 22°C under a 16 h light/8 h dark cycle (long day) or under an 8 h light/16 h dark cycle (short day) in a growth chamber.

### Construction of Recombinant Plasmids

To produce His_6_-FLC and GST-HPY2, full-length *FLC* and *HPY2* cDNAs were amplified by PCR and cloned into the pET28a (Novagen) and pGEX4T-1 (Amersham Biosciences) vectors, respectively. To prepare GST-FLC-Myc, full-length FLC cDNA was amplified using primers to add a Myc tag and cloned into pGEX4T-1. The FLC mutant proteins GST-FLCm1(K5R)-Myc, GST-FLCm2(K135R)-Myc, and GST-FLCm3(K154R)-Myc were prepared as previously described (Son et al., 2014). Numbers indicate the positions of the lysine (K) residues, and R indicates replacement of lysine with arginine residues. For production of His_6_-AtSUMO1, *Arabidopsis* full-length *SUMO1* cDNA was amplified by PCR and cloned into pET28a. Primer sequences are described in Supplementary Table S1.

### Purification of Recombinant Proteins

All recombinant proteins were expressed in *Escherichia coli* strain BL21 and purified as previously described (Colby et al., 2006; Son et al., 2014). Briefly, His_6_-AtSUMO1, His_6_-FLC, SUMO-activating enzyme E1 (His_6_-AtSAE1b and His_6_-AtSAE2), and SUMO-conjugating enzyme E2 (His_6_-AtSCE1) were purified with Ni^2+^-nitrilotriacetate (Ni^2+^-NTA) resins (Qiagen). GST, GST-FLC-Myc, GST-FLCm1(K5R)-Myc, GST-FLCm2(K135R)-Myc, GST-FLCm3(K154R)-Myc, and GST-HPY2 were purified with glutathione resins (Pharmacia).

### *In Vitro* and *In Vivo* Interaction Assays

*In vitro* pull-down assays to assess interactions between GST-HPY2 and His_6_-FLC were carried out using 2 μg each of GST-HPY2 bait and His_6_-FLC prey as previously described (Son et al., 2014). His_6_-FLC was detected by Western blot analysis using anti-His antibody (Santa Cruz Biotechnology).

Plant expression plasmids were constructed to investigate the direct interaction between HPY2 and FLC *in vivo*. To express FLC, the corresponding full-length cDNA was amplified using a forward primer to add a hexameric Myc (Myc_6_) and a reverse primer and cloned into the pBA002 vector. To express HPY2, the corresponding full-length cDNA was amplified by PCR using a forward primer and a reverse primer to add a FLAG_3_ tag and cloned into the pBA002 vector. Hexameric Myc-tagged or trimeric FLAG-tagged primers were synthesized by DNA synthesizer (Bioneer). Wild-type *Arabidopsis* plants were infiltrated with different combinations of *Agrobacterium* transformed with *35S-Myc_6_-FLC* or *35S-HPY2-FLAG_3_* constructs. Total protein was extracted from each sample 2 days after infiltration, and Myc_6_-FLC and HPY2-FLAG_3_ were detected by Western blot analysis with anti-Myc and anti-FLAG antibodies, respectively. Finally, total protein was extracted from each sample and immunoprecipitated with anti-FLAG antibody (1 μg/mL, Santa Cruz Biotechnology) in a buffer containing 50 mM Tris-Cl (pH 8.0), 150 mM NaCl, 10% glycerol, 1% NP-40, 2 mM EDTA, 1 mM PMSF, and a protease inhibitor cocktail (Promega). FLC was detected in immunoprecipitated samples by Western blot analysis with anti-Myc antibody (0.5 mg/mL, Sigma-Aldrich).

### Sumoylation Assays

*In vitro* sumoylation was performed in 30 μL of reaction buffer [200 mM Hepes (pH 7.5), 5 mM MgCl_2_, 2 mM ATP] with 50 ng of His_6_-AtSAE1b, 50 ng of His_6_-AtSAE2, 50 ng of His_6_-AtSCE1, 8 μg of His_6_-AtSUMO1, and 100 ng of GST-FLC-Myc, with or without 200 ng of GST-HPY2. After incubation for 3 h at 30°C, the reaction mixtures were separated on 11% SDS-PAGE gels. Sumoylated GST-FLC-Myc was detected by Western blot analysis using anti-Myc antibody (Santa Cruz Biotechnology). Sumoylation reactions were also performed as described above to identify the lysine residue modified by SUMO using WT FLC (GST-FLC-Myc) and mutant FLC [GST-FLCm1(K5R)-Myc, GST-FLCm2(K135)-Myc, or GST-FLCm3(K154R)-Myc].

### Preparation of Single and Double Transgenic Plants

Flowering Locus C- or mFLC(K154R)-overexpressing plants were prepared by floral dipping as previously described (Clough and Bent, 1998; Son et al., 2014). Double transgenic *Arabidopsis* plants overexpressing HPY2 and FLC or HPY2 and mFLC were produced by introduction of *XVE-HA_3_-HPY2* and *35S-FLC-FLAG_3_*, or *XVE-HA_3_-HPY2*, and *35S-mFLC-FLAG_3_*. Recombinant plasmid *XVE-HA_3_-HPY2* was generated by amplification of HPY2 cDNA with a forward primer tagged with trimeric HA_3_ (HA_3_) and a reverse primer, and insertion of the resultant fragment into the pET8 vector. Trimeric HA-tagged primers were synthesized by DNA synthesizer (Bioneer).

### Examination of HPY2 Effect on FLC Stability *In Vivo*

Two independent transgenic *35S-FLC-FLAG_3_* and *XVE HA_3_-HPY2* lines and two independent transgenic *35S-mFLC-FLAG_3_* and *XVE HA_3_-HPY2* lines were used for the experiment. Two-weeks-old double transgenic plants carrying *XVE HA_3_-HPY2* and *35S-FLC-FLAG_3_*, or *XVE HA_3_-HPY2*, and *35S-mFLC-FLAG_3_*, were treated with 10 μM β-estradiol. After 15 h, samples were ground in liquid nitrogen, and lysates were separated by 11% SDS-PAGE. FLC-FLAG_3_ and mFLC-FLAG_3_ levels were examined by Western blot analysis with anti-FLAG antibody. HA_3_-HPY2 induction was analyzed by Western blot analysis with anti-HA antibody.

### Examination of Flowering Time in *hpy2-2* Mutants

Flowering time was investigated under long- and short-day conditions. WT and *hpy2-2* mutant plants were grown, and rosette leaves were counted when inflorescences appeared. Twenty plants of each type (WT or *hpy2-2* mutant) were used in the experiment.

### Quantitative Real-time RT-PCR Analysis

Wild type and *hpy2-2* mutants were grown in long- and short-day conditions as described above. Total RNA was extracted from the leaves of WT and *hpy2-2* mutants, and quantitative real-time RT-PCR was used to assess transcript levels of *FLC*, *SUPPRESSOR OF OVEREXPRESSION OF CO 1* (*SOC1*), *FLOWERING LOCUS T* (*FT*), and *TWIN SISTER OF FT* (*TSF*) as described previously (Park et al., 2011). Tubulin amplification was used as an internal control. All reactions were repeated three times. Primer sequences are listed in Supplementary Table S1.

## Results

### FLC Interacts with HPY2 *In Vitro* and *In Vivo*

**FIGURE 1:**
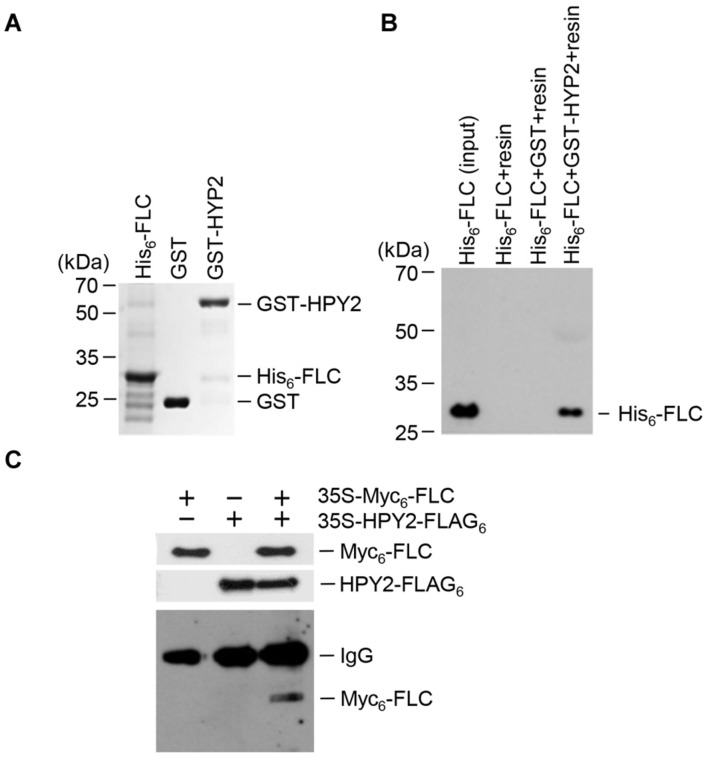
**Interaction of HPY2 with FLC.** **(A)** His_6_-FLC and GST-HPY2 were overexpressed in *Escherichia coli* and purified with Ni^2+^-NTA or glutathione affinity columns. **(B)** The His_6_-FLC protein was pulled down with the GST-HPY2 protein, separated on 11% SDS-polyacrylamide gels, and analyzed by Western blotting with anti-His antibody. **(C)** *In vivo* interaction of HPY2 and FLC. Wild-type (WT) plants were infiltrated with different combinations of *35S-Myc_6_-FLC* or *35S-HPY2-FLAG_3_* constructs. Total protein was extracted from each sample and immunoprecipitated with anti-FLAG antibody. After immunoprecipitation, Myc_6_-FLC was detected by Western blotting with anti-Myc antibody. Myc_6_-FLC and HPY2-FLAG_3_ expression was also examined by Western blotting with anti-Myc and anti-FLAG antibodies, respectively.

Previous results showed that E3 SUMO ligase AtSIZ1 interacted with FLC to inhibit sumoylation (Son et al., 2014). Here, we aimed to identify the E3 SUMO ligase that stimulated FLC sumoylation. HPY2, an *Arabidopsis* E3 SUMO ligase possessing an SP-RING (SIZ/PIAS-RING) domain, was chosen as a candidate. An *in vitro* pull-down assay was used to examine the interaction of HPY2 with FLC. Recombinant proteins GST-HPY2 and His_6_-FLC were overexpressed in *E. coli* and purified with glutathione or Ni^2+^-NTA resins (**Figure 1A**). The results showed that GST-HPY2 was able to pull down His_6_-FLC (**Figure 1B**). The interaction between HPY2 and FLC was also examined by immunoprecipitation (IP). Two constructs, *35S-Myc_6_-FLC* and *35S-HPY2-FLAG_3_*, were coinfiltrated into the leaves of WT plants. After IP with anti-FLAG antibody, Myc_6_-FLC was examined by Western blot analysis using anti-Myc antibody. Myc_6_-FLC was clearly detected (**Figure 1C**), indicating a strong interaction between HPY2 and FLC, consistent with the results of the *in vitro* pull-down experiment.

### FLC Sumoylation Is Stimulated by HPY2

**FIGURE 2:**
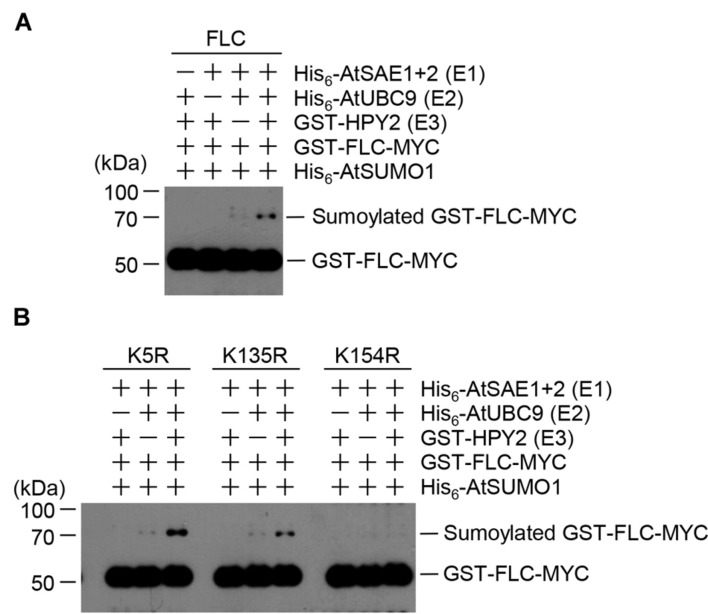
**Flowering Locus C (FLC) is sumoylated by HPY2 *in vitro*.** *Arabidopsis* His_6_-AtSAE1b, His_6_-AtSAE2, His_6_-AtSCE1, GST-HPY2, His_6_-AtSUMO1, and GST-FLC-Myc were overexpressed in *E. coli* and purified with Ni^2+^-NTA or glutathione affinity columns as appropriate. **(A)** Sumoylation of GST-FLC-Myc was assayed in the presence or absence of E1 (His_6_-AtSAE1b and His_6_-AtSAE2), E2 (His_6_-AtSCE1), E3 (GST-HPY2), and His_6_-AtSUMO1. FLC sumoylation was detected by Western blotting with anti-Myc antibody. **(B)** To identify the sumoylation site on FLC, GST-FLCm1-Myc (K54R), GST-FLCm2-Myc (K135R), and GST-FLCm3-Myc (K154R) were overexpressed in *E. coli* and purified using a glutathione affinity column. The reaction mixture contained E1 (His_6_-AtSAE1b and His_6_-AtSAE2), E2 (His_6_-AtSCE1), E3 (GST-HPY2), and His_6_-AtSUMO1 without (-) or with (+) a mutant protein instead of GST-FLC-Myc. FLC sumoylation was detected by Western blotting with anti-Myc antibody.

The strong interaction between HPY2 and FLC (**Figures 1B,C**) suggested that FLC could be modified by SUMO through E3 SUMO ligase activity of HPY2. Therefore, we next tested whether HPY2 had E3 SUMO ligase activity for FLC. *In vitro* sumoylation reactions were performed using His_6_-AtSAE1b, His_6_-AtSAE2, His_6_-AtSCE1, His_6_-AtSUMO1, GST-HPY2, and GST-FLC-Myc. The amount of sumoylated GST-FLC-Myc was increased by HPY2 in an E1- and E2-dependent manner (**Figure 2A**).

Our previous study showed that FLC contained three possible sumoylation sites (Son et al., 2014). Single- or double-mutant derivatives with the mutations K154R, K5R/K135R, K5R/K154R, or K135R/K154R were produced, and their sumoylation was examined by *in vitro* sumoylation assays (Son et al., 2014). The results showed K154 to be the principal site of SUMO conjugation on FLC (Son et al., 2014). In the current study, HPY2-mediated sumoylation was examined in the mutant FLC proteins GST-FLCm1(K5R)-Myc, GST-FLCm2(K135)-Myc, and GST-FLCm3(K154R)-Myc. Consistent with the previous study, GST-FLCm3(K154R)-Myc was not sumoylated, even in the presence of HPY2, confirming that the lysine residue at position 154 was a true sumoylation site (**Figure 2B**).

### HPY2 Stabilizes FLC

**FIGURE 3:**
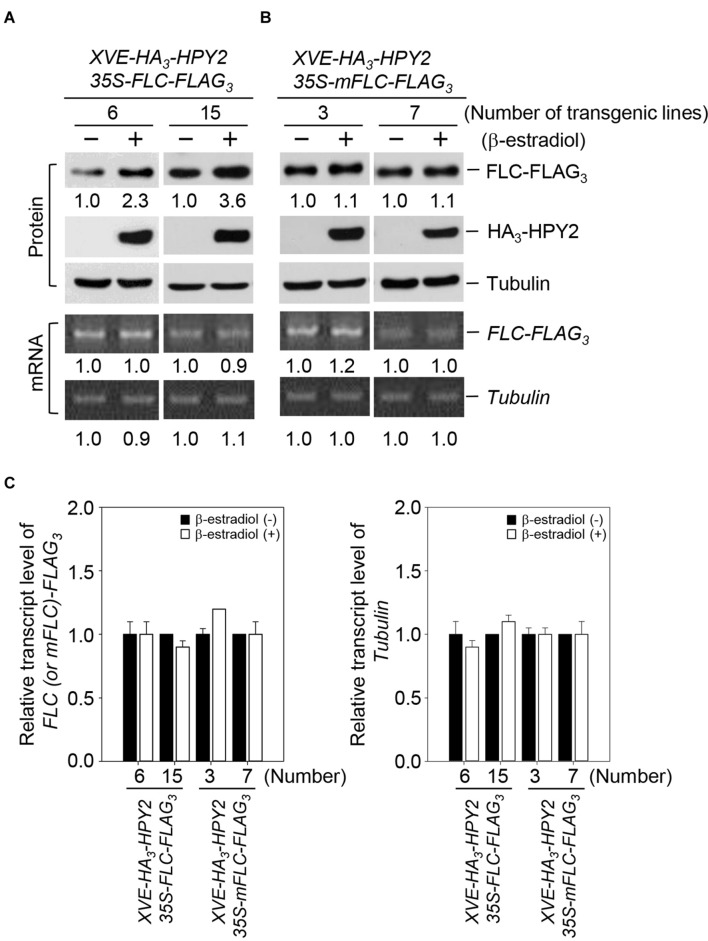
**Flowering Locus C is stabilized by HPY2 *in vivo*.** Double transgenic plants containing *35S-FLC-FLAG_3_* and *XVE-HA_3_-HPY2* **(A)** or *35S-mFLC (K154R)-FLAG_3_* and *XVE-HA_3_-AtSIZ1* **(B)** were incubated in liquid medium with β-estradiol to induce *HPY2* expression. After incubation for 15 h, HA_3_-HPY2, FLC-FLAG_3_, and mFLC-FLAG_3_ levels were assessed by Western blotting with anti-HA or anti-FLAG antibodies. Tubulin was used as a loading control. Numbers under lanes indicate relative intensities. Protein levels were normalized to a value of 1.00 for FLC or mFLC levels without inducer (“–” in both panels). RNA concentrations of *FLC-FLAG_3_* and *mFLC-FLAG_3_* were determined by real-time qRT-PCR using a FLAG primer and a gene-specific primer. *Tubulin* RNA was used as a loading control. **(C)** Graphical expression of *Tubulin*, *FLC-FLAG_3_*, and *mFLC-FLAG_3_* transcript levels from **(B)**. Bars indicate standard errors (*n* = 3).

Based on the strong interaction of HPY2 with FLC and the increase in FLC sumoylation as a result of HPY2 activity, we inferred that FLC stability could be modulated by HPY2. We therefore measured the effect of HPY2 on FLC levels using double transgenic plants with *XVE-HA_3_-HPY2* and *35S-FLC-FLAG_3_* or with *XVE-HA_3_-HPY2* and *35S-mFLC-FLAG_3_*. In mFLC, the lysine at position 154 (sumoylation site) was mutated to arginine (K154R). *HPY2* induction increased FLC levels by up to 2.3- and 3.6-fold in two independent transgenic plants (**Figures 3A,C**). However, no increase in mFLC level was seen after induction of *HPY2* in two independent transgenic plants (**Figures 3B,C**). *FLC* and *mFLC* transcript levels were similar after induction of *HPY2* expression, as determined using real-time qRT-PCR (**Figures 3A,B**). These data indicate that the FLC protein is stabilized by the E3 SUMO ligase activity of HPY2.

### A *hpy2* Mutant Exhibits Early Flowering

**FIGURE 4:**
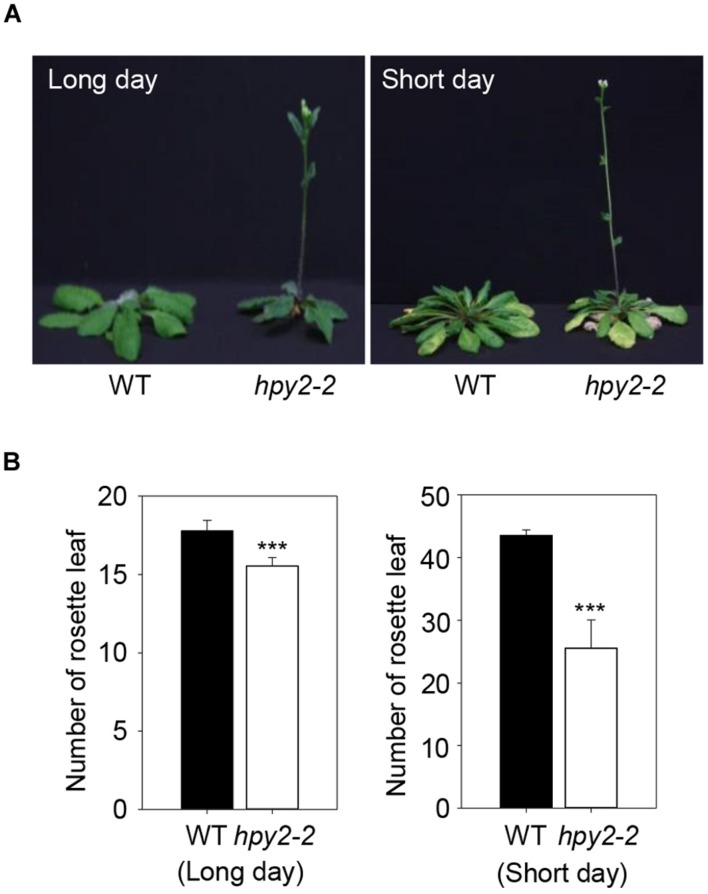
**Flowering time of *hpy2-2* mutants.** Early flowering was observed in the *hpy2-2* mutant grown under both long- and short-day conditions **(A)**. Flowering time was investigated by counting the number of rosette leaves present when inflorescences appeared **(B)**. The number of rosettes in WT and *hpy2-2* plants differed significantly (^∗∗∗^*P* < 0.0001, *t*-test, *n* = 20). Bars indicate standard errors.

Flowering Locus C is stabilized by HPY2-mediated sumoylation, suggesting that FLC functions may be compromised in a *hpy2* mutant background and that flowering may be affected. Two independent T-DNA insertion mutant lines, *hpy2-1* and *hpy2-2*, were characterized previously (Ishida et al., 2009, 2012). The *hpy2-2* mutant exhibits a relatively weak phenotype, but the *hpy2-1* mutant shows a severe growth defect phenotype and growth frequently stops prior to bolting. The *hpy2-2* mutant was therefore chosen to examine flowering time. Rosette leaves were counted just after bolting in WT and *hpy2-2* plants. Compared to WT, flowering was delayed in *hpy2-2* mutants under both long- and short-day conditions (**Figures 4A,B**). Flowering delay in *hpy2-2* was significant under short-day conditions but was less pronounced under long-day conditions (**Figures 4A,B**).

### Expression of Flowering-related Genes Is Affected by HPY2

**FIGURE 5:**
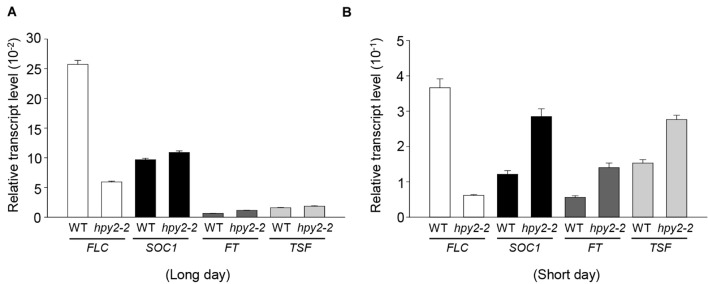
**Transcript levels of flowering-related genes in *hpy2-2* mutants.** Total RNA was isolated from the leaves of WT and *hpy2-2* mutant plants grown in soil under long **(A)**-or short-day **(B)** conditions. Transcript levels were examined using real-time qRT-PCR with gene-specific primers. Results are expressed as the mean ± SD (*n* = 3). *FLC*, *FLOWERING LOCUS C*; *SOC1*, *SUPPRESSOR OF OVEREXPRESSION OF CO 1*; *FT*, *FLOWERING LOCUS T*; *TSF*, *TWIN SISTER OF FT*.

HPY2 was identified as an E3 SUMO ligase for FLC, suggesting that the early flowering phenotype seen in *hpy2-2* mutants might be partly caused by low levels of FLC and decreased FLC activity. However, it was also possible that early flowering in *hpy2-2* mutants occurred as a consequence of altered gene expression. To investigate this, real-time qRT-PCR was used to measure transcript levels of *FLC* and the flowering-related genes *SOC1*, *FT*, and *TSF* in WT and *hpy2-2* mutants grown in long- or short-day conditions (**Figures 5A,B**). *SOC1*, *FT*, and *TSF* were slightly upregulated in *hpy2-2* mutants compared with their expression in WT plants under long-day conditions (**Figure 5A**). More substantial differences in the expression of *SOC1*, *FT*, and *TSF* were apparent under short-day conditions, with approximately twofold higher transcript levels observed in *hpy2-2* plants compared to WT plants (**Figure 5B**).

## Discussion

In this study, HPY2 was shown to be a functional E3 SUMO ligase that was able to directly interact with FLC to catalyze its sumoylation.

To date, two SIZ-type E3 SUMO ligases have been identified in plants, namely, AtSIZ1 and HPY2. The functional roles and phenotypic features of AtSIZ1 are well-characterized. However, the functions of HPY2 are poorly understood. A recent study showed that HPY2 had self-sumoylation activity *in vitro*, and that mutation of HPY2 reduced SUMO-conjugate levels in *Arabidopsis* (Ishida et al., 2009). In addition, loss of HPY2 function resulted in a premature mitotic-to-endocytic transition that led to severe dwarfism (Ishida et al., 2009). Whereas the phenotype of *hpy2-1* mutants did not depend on the accumulation of salicylic acid (SA), the *siz1-2* mutant phenotype was caused by SA accumulation, indicating that the two proteins functioned through different pathways and that their roles did not overlap (Ishida et al., 2012).

Sumoylation of target proteins by AtSIZ1 is well-characterized (Elrouby and Coupland, 2010; Miller et al., 2010; Miura and Hasegawa, 2010; Park et al., 2011; Zheng et al., 2012; Conti et al., 2014; Son et al., 2014; Kim D.Y. et al., 2015; Kim S.I. et al., 2015). However, the targets of HPY2 remain unidentified, and E3 ligase activity of HPY2 against target proteins has not been reported to date. In a previous study, we showed that AtSIZ1 interacts directly with FLC and stabilizes the protein (Kim S.I. et al., 2015). However, our results showed that AtSIZ1 inhibited FLC sumoylation, which led us to speculate that HPY2 may act as a specific E3 SUMO ligase for FLC. An *in vitro* sumoylation assay showed that HPY2 had E3 SUMO ligase activity against FLC through direct interaction (**Figures 1B,C** and **2A**). This is the first report demonstrating HPY2 E3 SUMO ligase activity for a target protein.

Modification of target lysines by SUMO occurs by two different mechanisms. In the first mechanism, target proteins are directly modified by conjugating enzyme E2 (Bernier-Villamor et al., 2002; Meulmeester et al., 2008; Zhu et al., 2008). The second mechanism involves E3 ligase-mediated sumoylation (Park and Yun, 2013). Our previous study showed that FLC could be sumoylated without the help of an E3 SUMO ligase, and that FLC sumoylation was inhibited by the E3 SUMO ligase AtSIZ1. In the present study, we found that the level of sumoylated FLC was increased by the addition of HPY2 (**Figure 2A**), indicating that FLC sumoylation was stimulated by HPY2 via its E3 SUMO ligase activity.

Our previous study showed that AtSIZ1 inhibited FLC sumoylation but stabilized the FLC protein (Son et al., 2014). In the current study, HPY2 overexpression led to an increase in levels of FLC but not in levels of mFLC, which lacked the critical lysine residue for sumoylation (**Figures 3A–C**), indicating that FLC sumoylation by HPY2 stabilized FLC. In yeast, animal, and plant systems, modification by SUMO affects the stability and activity of target proteins (Park and Yun, 2013). Consistent with this, our results showed that sumoylation had a positive effect on FLC function.

Since FLC is a central regulator of flowering and HPY2 has E3 SUMO ligase activity against FLC, we next examined flowering time in the *hpy2-2* mutant. Mutant plants flowered earlier than WT plants (**Figure 4**). Previously, we reported that flowering time was significantly delayed by the overexpression of WT *FLC* but that flowering time was unaffected by overexpression of *mFLC* (Son et al., 2014), indicating that sumoylation was critical for the floral repressor function of FLC. This suggests that early flowering in *hpy2-2* mutants is caused by the reduced stability and lower activity of FLC resulting from the loss of HPY2 E3 SUMO ligase activity.

Our data suggest that early flowering in the *hpy2-2* mutant results from the loss of HPY2 activity, which leads to low FLC levels or low FLC activity. However, it was possible that early flowering of the *hpy2-2* mutant might be a consequence of changes in gene expression. *FLC* transcript levels were decreased in the *hpy2-2* mutant (**Figure 5**) indicating that HPY2 regulated FLC-mediated flowering at the transcriptional level as well as at the post-translational level.

## Conclusion

HPY2 controls FLC-mediated floral transition through its E3 SUMO ligase activity as well as through direct binding to FLC. Taken together with previous findings, our data indicate that two E3 SUMO ligases, AtSIZ1 and HPY2, are involved in the regulation of FLC stability, and that sumoylation is a critical modification for the regulation of FLC function. Elucidation of the mechanisms underlying the regulation of FLC function by AtSIZ1 and HPY2, alone or in combination, will enhance our understanding of the modulation of flowering time by the SUMO system.

## Author Contributions

HS designed the project. JK, GS, and S-IK carried out experiments. JK, GS, S-IK, JS, and HS analyzed and interpreted the data. JK and HS wrote the manuscript. All authors commented on the results and the manuscript.

## Conflict of Interest Statement

The authors declare that the research was conducted in the absence of any commercial or financial relationships that could be construed as a potential conflict of interest.
