# Supplementary material for: Arabidopsis HIGH PLOIDY2 Sumoylates and Stabilizes Flowering Locus C through Its E3 Ligase Activity
Source: Front Plant Sci. 2016 Apr 20;7:530. doi: 10.3389/fpls.2016.00530 (PMC4837325; doi:10.3389/fpls.2016.00530)
Supplement: Supplementary file 1 [file Table_1.DOCX]

**Supplementary Table 1. List of primers used in this study.**

| Purpose | gene | Forward primer | Reverse primer |
| --- | --- | --- | --- |
| Real time PCR | FLC | 5’-CGGTCTCATCGAGAAAGCTC-3’ | 5’-CCACAAGCTTGCTATCCACA-3’ |
|  | SOC1 | 5’-AATTCGCCAGCTCCAATATG-3’ | 5’-ATCTGTTGCAGCTCCTCGAT-3’ |
|  | FT | 5’-CTGGAACAACCTTTGGCAAT-3’ | 5’-AGCCACTCTCCCTCTGACAA-3’ |
|  | TSF | 5’-CAACCCTCACCAACGAGAAT-3’ | 5’-ACCGTTTGTCTTCCGAGTTG-3’ |
|  | TUB | 5’-CGAAAACGCTGACGAGTGTA-3’ | 5’-CCTTGGGAATGGGATAAGGT-3’ |
| Production of transgenic plant | FLC-FLAG_3_ | 5’-TCTACCTCGAGATGGGAAGAAAAAAACTAGAAATC-3’ | 5’-TCTACGAGCTCTCACTTGTCATCGTCATCCTTGTAGTCCTTGTCA TCGTCATCCTTGTAGTCCTTGTCATCGTCATCCTTGTAGTCCATAT-3’ |
|  | HPY2-HA_3_ | 5’-CTCGAGATGGCGTCGG CGTCCTCGTC-3’ | 5’-ACTAGTAGCTAATCTTCATCCACATCTT-3’ |
|  | HIS_6_-FLC | 5’- ATGCAGAATTCATGGGAAGAA AAAAACTAGA AA-3' | 5’- ATGCACTCGAGATTAAGTAGTGGGAGAGTCACC-3’ |
| Pull down  assay | GST-HPY2 | 5’-GAATTCCTAATCTTCATCCACATCTT-3’ | 5’-GTCGACACTAATCTTCATCCACATCTT-3’ |
|  | MYC_6_-FLC | 5’-TGGATCCGTATGGGAAGAA AAAAACTAGA AA-3’ | 5’-ACTAGTGATTAAGTAGTGGGAGAGTCACC-3’ |
|  | HPY2-FLAG_6_ | 5’-TGGATCCGTCTAATCTTCATCCACATCTT-3’ | 5’-ACTAGTGCTAATCTTCATCCACATCTT-3’ |
| *In vitro* sumoylation  assay | GST-FLC-MYC | 5’-TCTACGAATTCATGGGAAGAAAAAAACTA-3' | 5’-TCTACCTCGAGTTATTCATTCAAGTCCTCTTCAGAAATGAGCTTT TGCTCCATATTAAGTAGTGGGAGAGT-3’ |
